# Supplementary material for: The Synthesis and Evaluation of RGD−Conjugated Chitosan Gel as Daily Supplement for Body Weight Control
Source: Materials (Basel). 2021 Aug 10;14(16):4467. doi: 10.3390/ma14164467 (PMC8399670; doi:10.3390/ma14164467)
Supplement: Supplementary file 1 [file materials-14-04467-s001.zip › materials-1315469-supplementary.pdf]

materials

MDPI

Supplementary Material

The Synthesis and Evaluation of RGD-Conjugated Chitosan Gel as Daily Supplement for Body Weight Control

Wei-Yao Chen [1], Yu-Ting Chen [2], Cherng-Jyh Ke [3], Ching-Yun Chen [4] and Feng-Huei Lin [2,5,*]

1 Institute of Biotechnology, National Taiwan University, Taipei 106216, Taiwan; tp6jo3ul6@hotmail.com
2 Institute of Biomedical Engineering, National Taiwan University, Taipei 106216, Taiwan; tingamy93@gmail.com
3 Biomaterials Translational Research Center, China Medical University Hospital, Taichung 40202, Taiwan; fonchanwd@gmail.com
4 Department of Biomedical Sciences & Engineering, National Central University, Taoyuan 32001, Taiwan; chingyun523@gmail.com
5 Institute of Biomedical Engineering and Nanomedicine, National Health Research Institutes, Miaoli County 35053, Taiwan
* Correspondence: double@ntu.edu.tw

Citation: Chen, W.-Y.; Chen, Y.-T.; Ke, C.-J.; Chen, C.-Y.; Lin, F.-H. The Synthesis and Evaluation of RGD–Conjugated Chitosan Gel as Daily Supplement for Body Weight Control. *Materials* **2021**, *14*, 4467. https://doi.org/10.3390/ma14164467

Academic Editor: Joaquim Miguel Oliveira, Viviana Punto Ribeiro, Rui L. Reis

Received: 11 July 2021
Accepted: 2 August 2021
Published: 10 August 2021

Publisher's Note: MDPI stays neutral with regard to jurisdictional claims in published maps and institutional affiliations.

Copyright: © 2021 by the authors. Submitted for possible open access publication under the terms and conditions of the Creative Commons Attribution (CC BY) license (http://creativecommons.org/licenses/by/4.0/).

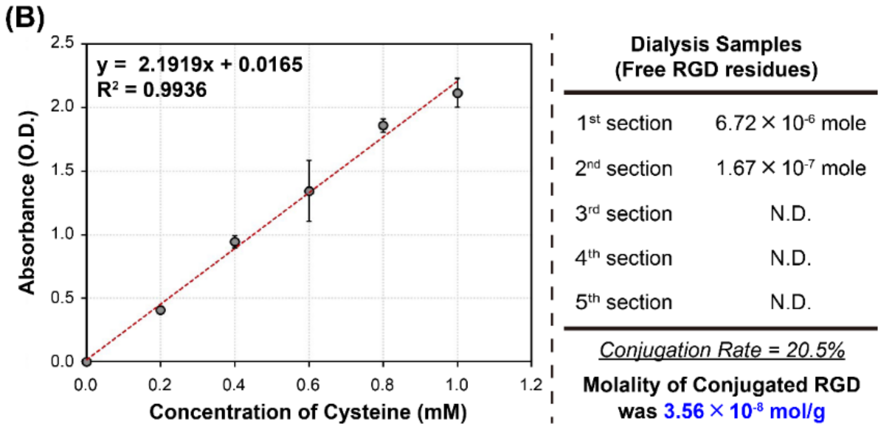

Figure S1. Ninhydrin test concentration calculation of the RGD peptide, RGD-Chitosan conjugate, and 90% deacetylated chitosan.

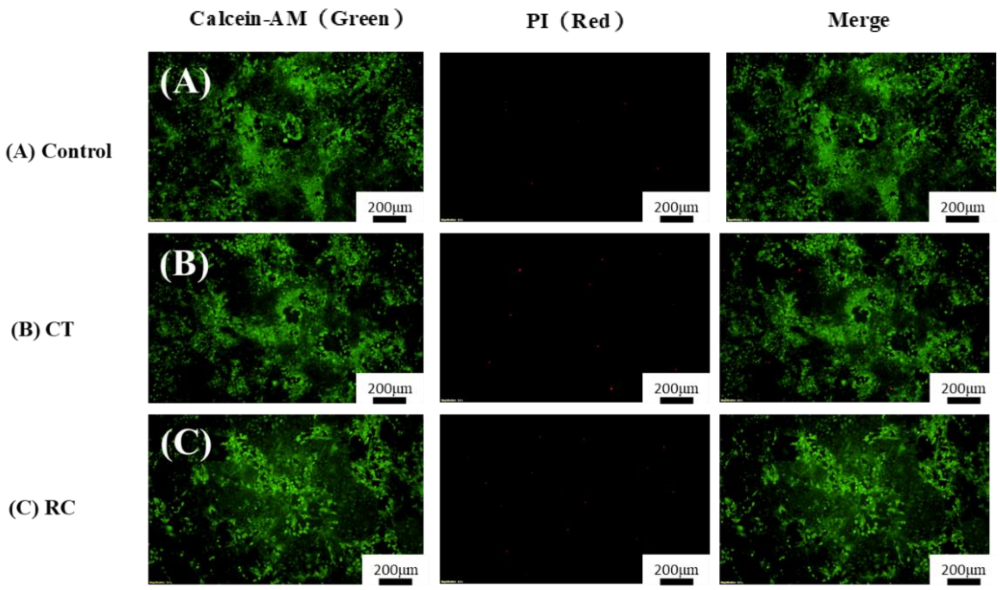

Figure S2. Live and dead staining. (**A**) Control; (**B**) Chitosan (CT), and (**C**) RGD-Chitosan conjugate (RC).

Table S1. Blood and hematic biometrics of the normal control (NC), chitosan (CT) and RGD-Chitosan conjugate (RC) groups.

	**NC**	**CT**	**RC**
WBC(10^3/μL)	9.14	13.57	11.40
N E%	3.05	13.85	13.19
LY%	5.71	86.35	82.57
MO%	0.25	3.39	3.77
EO%	0.11	0.59	0.75
BA%	0.02	0.48	0.43
RBC (10^6/μL)	14.03	8.01	7.12
HGB (g/dl)	79.85	15.97	13.83
HCT (%)	2.36	46.47	40.83
MCV (fl)	1.17	57.67	56.77
MCH (pg)	0.40	20.03	19.13
MCHC (g/dl)	7.45	34.37	33.73
RDW (%)	15.17	13.80	14.87
PLT (10^3/μL)	42.03	905.67	836.67
MPV (fl)	56.50	6.67	6.40
GLU/BIL/KET	Negative	Negative	Negative
SG	1.014	1.014	1.016
BLO/NIT/LEU	Negative	Negative	Negative
pH	6.5	7.0	7.0
PRO	Negative	Negative	Negative
URO	0.2	0.2	0.2
